# Draft Genome Sequence of Rhizobium ruizarguesonis (Rhizobium leguminosarum) Strain 1TK341

**DOI:** 10.1128/mra.01023-21

**Published:** 2022-03-17

**Authors:** Alexey M. Afonin, Emma S. Gribchenko, Anton S. Sulima, Gulnar A. Akhtemova, Vladimir A. Zhukov

**Affiliations:** a All-Russia Research Institute for Agricultural Microbiology (ARRIAM), Laboratory of Genetics of Plant-Microbe Interactions, Saint Petersburg, Russia; b Sirius University of Science and Technology, Sochi, Russia; SIPBS, University of Strathclyde

## Abstract

Rhizobium ruizarguesonis (Rhizobium leguminosarum) strain 1TK341 was isolated from pink nodules of fixation-negative mutant line P61 of pea (Pisum sativum L.) grown in soil. Here, we report the draft genome sequence of the strain.

## ANNOUNCEMENT

**FIG 1:**
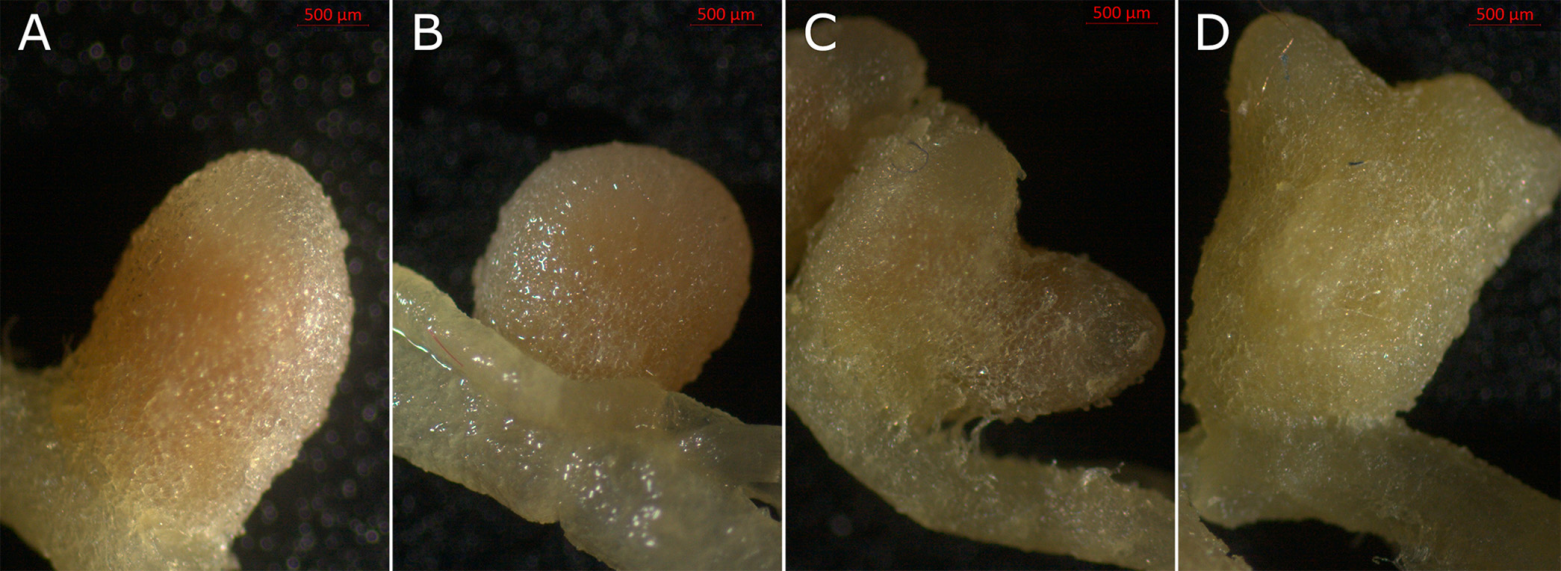
Phenotypes of the pea cv. Frisson (wild type) and pea line P61 (*sym25*) nodules in symbiosis with specific *Rhizobium* sp. strains. (A) Pink nodule formed in symbiotic pair cv. Frisson (wild type) and 1TK341; (B) pink nodule formed in symbiotic pair P61 (*sym25*) and strain RCAM1026; (C) pink nodule formed in symbiotic pairP61 (*sym25*) and strain 1TK341; (D) white nonfixing nodule formed in symbiotic pair line P61 (*sym25*) and strain 3841.

Strain 1TK341 was first isolated from the nodules of pea (Pisum sativum L.) line P61 plants cultivated in a pot filled with soil from the test field (59° 44′ 41.802 N, 30° 26′ 17.8548 E) of the All-Russia Research Institute for Agricultural Microbiology (ARRIAM), similarly to the previously described strain A1 (1, 2). The P61 line carries a mutation in the symbiotic gene *sym25*, forms white nodules (unable to fix N_2_) with most Rhizobium leguminosarum strains, but is able to form pink, nitrogen-fixing nodules with a reduced set of R. leguminosarum strains (3). The symbiotic phenotype of this strain and those of strains RCAM1026 (4) and 3841 (5) are shown in Fig. 1. This work describes the first assembly of the genome of 1TK341.

Plants of *P. sativum* line P61 were inoculated with the 1TK341 strain, and pink nodules were harvested. The nodules were surface sterilized with 98% ethanol, nodules were crushed, and the material was plated onto solid TY media (6). One colony was chosen for DNA isolation. The culture was cultivated in 50 mL of liquid TY medium in a 100-mL flask at 28°C and 200 rpm (7). The culture was harvested after 48 h of incubation. DNA was isolated using the phenol-chloroform method as described previously (8) and quantified with a spectrophotometer (BioSpec-mini; Shimadzu, Japan). The required absorption for library parameters were *A*_260_/*A*_280_ of ∼2 and *A*_260_/*A*_230_ of >1.8.

A library for short-read sequencing was prepared using the TruSeq DNA PCR-free kit (Illumina, Inc., San Diego, CA) and sequenced using an Illumina 2000 platform. In total, 3,498,009 2 × 150-bp reads were generated. The reads were quality trimmed, and adapter sequences were removed with the BBDuk tool from the BBMap (v.38.90) (9) package (ktrim=r k = 23 mink = 11 hdist = 1 tpe tbo minlen = 25 qtrim=rl trimq = 10); after filtering, the expected coverage was 95×. Unicycler (v.0.4.9) (10) was used to assemble the reads. The assembly consists of 109 contigs, with a cumulative length of 7,630,733 bp, *N*_50_ of 216628, and GC content of 60.76%. The Prokaryotic Genome Annotation Pipeline (PGAP; v.5.3) (11) was used to annotate the assembly. It contains 7,148 protein-coding genes, 1 rRNA operon, and 47 tRNA genes.

The assembly was compared to that of strains of R. leguminosarum species complex (12) using the fastANI (v.1.33) algorithm (13); the closest strains were JHI985 (ASM1066844v1) (average nucleotide identity [ANI], 98.9%), and RCAM1026 (ASM192726v3) (ANI, 98.6%), both belonging to genospecies C, which was recently renamed *Rhizobium ruizarguesonis* (14). This means that strain 1TK341 belongs to *R. ruizarguesonis*. Further investigation of this strain could possibly lead to the discovery of the mechanism behind the increased specificity of the interaction with the P61 line.

### Data availability.

The assemblies and sequence data have been uploaded to the NCBI database. The BioProject number is PRJNA768928, the BioSample number is SAMN22069953, and the GenBank accession number is JAJAEH000000000. The raw Illumina data are deposited under the accession number SRR16242825. This announcement describes the first version of the genome assembly.
